# Machine Learning and Symptom Patterns in Degenerative Cervical Myelopathy: Web-Based Survey Study

**DOI:** 10.2196/54747

**Published:** 2024-01-25

**Authors:** Alvaro Yanez Touzet, Tanzil Rujeedawa, Colin Munro, Konstantinos Margetis, Benjamin M Davies

**Affiliations:** 1 University of Manchester Manchester United Kingdom; 2 University of Cambridge Cambridge United Kingdom; 3 Icahn School of Medicine at Mount Sinai New York, NY United States

**Keywords:** cervical, myelopathy, machine learning, cluster, clusters, clustering, spine, spinal, compression, neck, degenerative, k-means, patient reported, degenerative cervical myelopathy

## Abstract

**Background:**

Degenerative cervical myelopathy (DCM), a progressive spinal cord injury caused by spinal cord compression from degenerative pathology, often presents with neck pain, sensorimotor dysfunction in the upper or lower limbs, gait disturbance, and bladder or bowel dysfunction. Its symptomatology is very heterogeneous, making early detection as well as the measurement or understanding of the underlying factors and their consequences challenging. Increasingly, evidence suggests that DCM may consist of subgroups of the disease, which are yet to be defined.

**Objective:**

This study aimed to explore whether machine learning can identify clinically meaningful groups of patients based solely on clinical features.

**Methods:**

A survey was conducted wherein participants were asked to specify the clinical features they had experienced, their principal presenting complaint, and time to diagnosis as well as demographic information, including disease severity, age, and sex. K-means clustering was used to divide respondents into clusters according to their clinical features using the Euclidean distance measure and the Hartigan-Wong algorithm. The clinical significance of groups was subsequently explored by comparing their time to presentation, time with disease severity, and other demographics.

**Results:**

After a review of both ancillary and cluster data, it was determined by consensus that the optimal number of DCM response groups was 3. In Cluster 1, there were 40 respondents, and the ratio of male to female participants was 13:21. In Cluster 2, there were 92 respondents, with a male to female participant ratio of 27:65. Cluster 3 had 57 respondents, with a male to female participant ratio of 9:48. A total of 6 people did not report biological sex in Cluster 1. The mean age in this Cluster was 56.2 (SD 10.5) years; in Cluster 2, it was 54.7 (SD 9.63) years; and in Cluster 3, it was 51.8 (SD 8.4) years. Patients across clusters significantly differed in the total number of clinical features reported, with more clinical features in Cluster 3 and the least clinical features in Cluster 1 (Kruskal-Wallis rank sum test: *χ*^2^_2_=159.46; *P*<.001). There was no relationship between the pattern of clinical features and severity. There were also no differences between clusters regarding time since diagnosis and time with DCM.

**Conclusions:**

Using machine learning and patient-reported experience, 3 groups of patients with DCM were defined, which were different in the number of clinical features but not in the severity of DCM or time with DCM. Although a clearer biological basis for the clusters may have been missed, the findings are consistent with the emerging observation that DCM is a heterogeneous disease, difficult to diagnose or stratify. There is a place for machine learning methods to efficiently assist with pattern recognition. However, the challenge lies in creating quality data sets necessary to derive benefit from such approaches.

## Introduction

Degenerative cervical myelopathy (DCM) is a progressive spinal cord injury caused by spinal cord compression from degenerative pathology and consists of various subcategories of pathology, including cervical spondylotic myelopathy, ossification of the posterior longitudinal ligament, ossification of the ligamentum flavum, and degenerative disc disease [1-4]. It is estimated to affect 2% of adults, although fewer than 10% are currently diagnosed [5,6]. Surgery is the mainstay of treatment for DCM, aiming to decompress the spinal cord [2,7-9].

DCM often presents with neck pain, sensorimotor dysfunction in the upper or lower limbs, gait disturbance, and bladder or bowel dysfunction [2,10-14]. Examination findings include upper motor neuron signs in the limbs, such as positive Babinski sign, positive Hoffman sign, hyperreflexia, and increased tone [2,10-13]. Its symptomatology is very heterogeneous, making early detection difficult. This heterogeneity makes it difficult to measure or understand what drives consequences. For instance, the heterogeneity has made it harder to understand health-related quality of life [15]. This has also hindered comparisons between studies and the development of clinical practice guidelines and recommendations for DCM [16,17]. Additionally, once detected, DCM is unpredictable due to a lack of reliable methods to determine prognosis.

Increasingly, evidence suggests that DCM may consist of subgroups of the disease, which still need to be defined [18-20]. Machine learning can help in finding them. In fact, machine learning has shown potential in predicting health-related quality of life after surgery for mild DCM and outcome after surgery, although external validation and prospective analysis are still needed [21,22]. The use of machine learning in identifying these subgroups is dependent on the data set. Munro et al [23] (2023) provide a unique and comprehensive description of the effects of DCM from the perspective of people living with DCM [24,25]. This is a data set that could lend itself to machine learning analysis due to its comprehensiveness.

The objective of this study was to explore whether machine learning can identify clinically meaningful groups of patients based on solely clinical features.

## Methods

### Data Set

A mixed methods cross-sectional study was conducted by a team from the University of Cambridge through Myelopathy.org [26], a global charity dedicated to DCM. A focus group session of people with DCM and their supporters was used to inform the development of a web-based survey to explore the consequences of living with DCM. The survey was advertised using the Myelopathy.org website, an international nonprofit organization dedicated to promoting understanding and awareness of DCM. Survey participants were asked to specify the clinical features they had experienced, their principal presenting complaint, and time to diagnosis as well as demographic information, including disease severity, age, and sex. The data consist of 189 yes or no responses to a list of 76 clinical features. This was published in a paper, titled “Targeting earlier diagnosis: what symptoms come first in degenerative cervical myelopathy?” [23], wherein the full methodology is detailed.

### Analysis

Patients were grouped into subsets with similar characteristics using k-means clustering. K-means clustering is a method that groups data into “k” nonoverlapping, distinct subsets by finding centroids in the data representing each cluster’s center and allocating data points to each cluster by minimizing within-cluster variance around centroids. K-means clustering was used due to its efficiency for small data sets and explainability, aiming to group respondents into clusters based on their clinical features, using the Euclidean distance measure and the Hartigan-Wong algorithm [27]. The optimal number of clusters (k) was determined through the inspection of 3 ancillary methods, namely, the elbow, silhouette, and gap statistic methods [28]. The clinical significance of groups was subsequently explored by comparing their time to presentation, time with disease severity, and other demographics. DCM severity was assessed using total Modified Japanese Orthopaedic Association scores [29]. Noncomplete records were not excluded, and missing data were not imputed. All analyses were conducted in R (version 4.1.0; R Foundation for Statistical Computing) [30].

### Ethical Considerations

This study was conducted with ethical approval from the University of Cambridge (HBREC.2019.14). At the start of the survey, participants were provided with an overview of the study and definition of DCM, and by continuing into the survey, participants were confirming their diagnosis of DCM and providing informed consent to participate. All data collected were anonymous. No incentives were offered for the completion of the surveys.

## Results

### Cohort Demographics

Of the 189 participants, 134 were female and 49 were male (6 did not report biological sex). Respondents were on average 54.1 years of age. A total of 29 of them had mild DCM, 68 had moderate DCM, and 92 had severe DCM. The majority (131/189, 69%) reported having had surgery for DCM.

### Cluster Analysis

**Figure 1:**
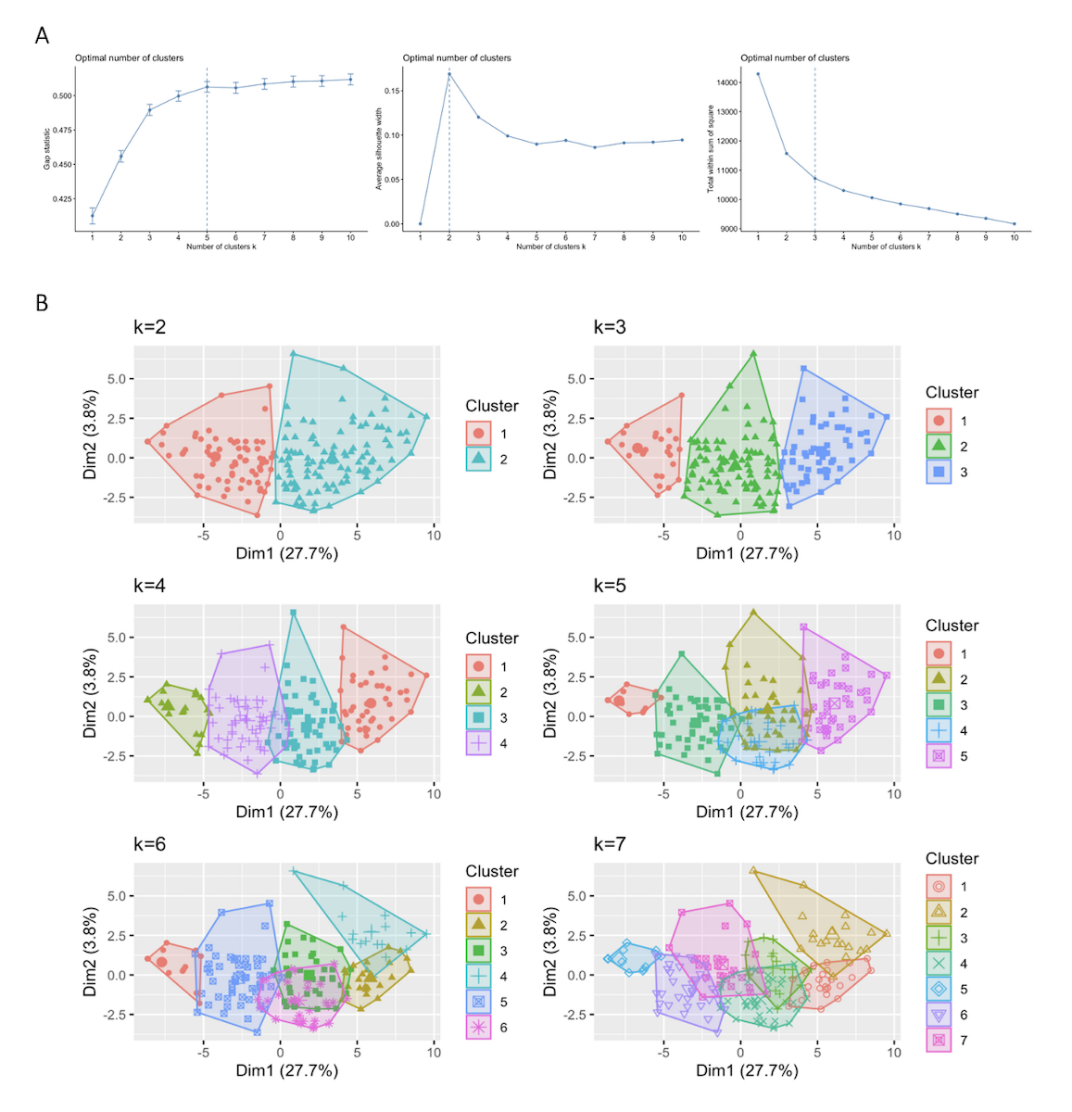
(A) Determining the optimal number of clusters; (B) k-means clustering (euclidean).

Ancillary methods suggested different optimal numbers of clusters (k). Elbow, silhouette, and gap statistic methods identified k=3, k=2, and k=5, respectively (Figure 1A). The data were hence clustered into multiple values of k and inspected (Figure 1B). After a review of both ancillary and cluster data, it was determined by consensus between AYT and BD that the optimal number of DCM response groups was 3. The reasoning behind this was that the ancillary curves in 2 out of 3 ancillary methods plateaued from k≥3 (Figure 1A), but clusters above k≥4 overlapped (Figure 1B).

### Characterization Analysis

In Cluster 1, there were 40 respondents, and the ratio of male to female participants was 13:21. In Cluster 2, there were 92 respondents, with a male to female participant ratio of 27:65. Cluster 3 had 57 respondents, with a male to female participant ratio of 9:48. A total of 6 people did not report biological sex in Cluster 1. The mean age was 56.2 (SD 10.5) years in this cluster; in Cluster 2, it was 54.7 (SD 9.63) years; and in Cluster 3, it was 51.8 (SD 8.4) years.

**Figure 2:**
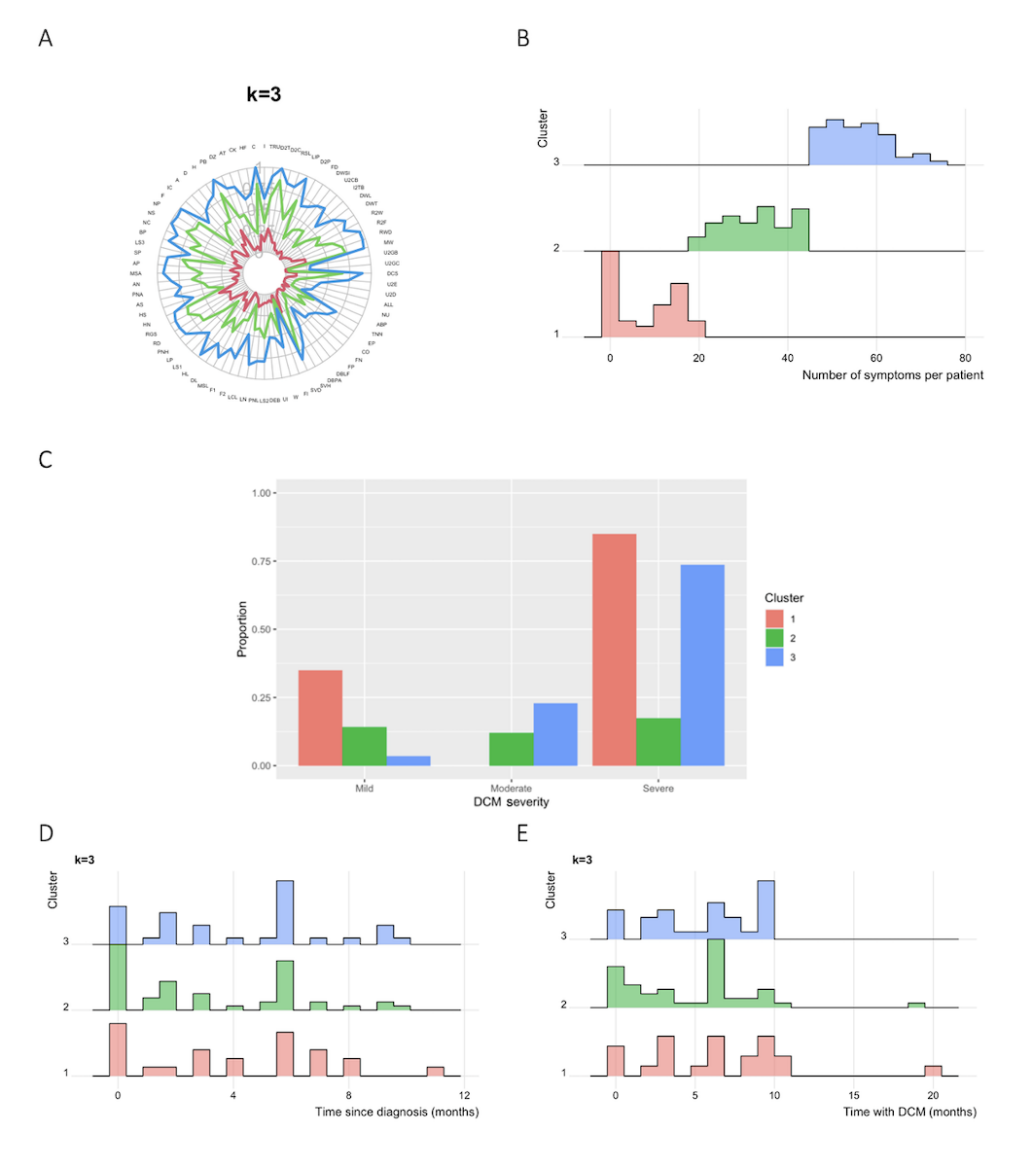
(A) Spider charts showing survey responses across clusters (the abbreviations along the circumference are detailed in the table in the Multimedia Appendix 1); the radius represents relative frequency, normalized to 1; (B) total number of clinical features reported across clusters; (C) proportions of degenerative cervical myelopathy (DCM) severity across clusters (based on the Modified Japanese Orthopaedic Association scores); (D) distribution of time taken to be diagnosed with DCM in each cluster; (E) distribution of time with DCM in each cluster.

A spider chart was subsequently generated to explore the clinical significance of the clusters, wherein the curves did not cross (Figure 2A; Multimedia Appendix 1).

It was also checked if patients in the different groups experienced differing numbers of features (Figure 2B). Patients across clusters significantly differed in the total number of clinical features reported, with more clinical features in Cluster 3 and the least clinical features in Cluster 1 (Kruskal-Wallis rank sum test: *χ*^2^_2_=159.46; *P*<.001).

To check whether patients with more clinical features had a more severe form of DCM, patterns of clinical features against severity were compared. The results showed no relationship between the pattern of clinical features and severity (Figure 2C).

Patterns of clinical features against both time since diagnosis and time with DCM were also analyzed. As shown in Figures 2D and 2E, there did not seem to be any differences between clusters in these distributions.

## Discussion

### Principal Findings

Cluster analysis suggested 3 optimal subgroups based on clinical features. When exploring why these groups differed in terms of cohort demographics, only the number of reported symptoms differed significantly. The pattern of clinical features within each of the 3 groups was similar. Notably, the 3 curves in the spider chart appear to peak and trough in a similar pattern, suggesting that there was no difference in the pattern of clinical features. The concentricity of curves, however, suggested that clustering may be due to the total number of features experienced. This possibility was statistically significant (Kruskal-Wallis rank sum test). Finally, there was no link between the groups and disease severity, time with DCM, and time since diagnosis.

### Limitations

This study has several limitations. The data represent a single time point cross-sectional survey of an internet-recruited cohort of patients, which could limit the generalizability of the findings. Additionally, information on disease characteristics, used for exploring the clinical significance of clusters, was limited to time with symptoms and a self-reported modified Japanese Orthopaedic Association score [31]. A more diverse data set would be more insightful, especially in DCM, wherein the nuances of symptom presentation and progression are critical. The sample size is also relatively small by machine learning standards. Finally, only 1 analysis method (k-means clustering) was performed, which may prevent us from capturing the full complexity of DCM symptomatology, especially with the increasing prominence of personalized approaches [32].

That being said, this is a unique data set, formed from the unrestricted perspectives of almost 200 patients; it was formed without any preconceptions regarding what symptoms were considered related to DCM. The result is also not unexpected. Standard analytical approaches, using more traditional data sets, have failed to stratify patients by symptoms [33]. Consequently, although a clearer biological basis for the clusters may have been missed, the findings are consistent with the emerging observation that DCM is a heterogeneous disease, difficult to diagnose or stratify [15-17]. This has been highlighted by the work of Cook et al [34] (2022) and is perhaps reflected in our inability to explain the variability in the quality of life in DCM [15].

This study shows that there is certainly a role for machine learning methods to efficiently assist with pattern recognition, but data sets must be large, valid, and comprehensive. In DCM, the challenge and priority appear to be less focused on data set size and more focused on the type of data [35]. For example, our redefinition of DCM in terms of time, mechanical stress, and vulnerability to sustain a spinal cord injury has highlighted the potential significance of various disease factors; these factors range from frailty and genetics to the type of pathology causing compression, encompassing the likely heterogeneous mechanical loading they induce [20]. Further, there are few valid and reliable outcome measures available, with most relying on face-to-face presentations to measure changes over the course of months, exhibiting low statistical power. The work of Cook et al [34] (2022) has highlighted that the experience of DCM is driven by social determinants—features such as ethnicity as well as educational, and economic status [34]. This means subjectivity in outcomes will drive current variability. Novel biomarkers, including imaging, blood, and digital biomarkers, are likely to hold value in this context, offering more disease-specific and sensitive disease indicators [36]. The need for more comprehensive and improved measurement is a firm priority in DCM [16]. Therefore, artificial intelligence undoubtedly has an important role in the future of DCM research and care. To our knowledge, such measures do not currently exist. Analysis of one of the most detailed cohorts also failed to identify biologically significant strata [22,37]. Therefore, the short-term challenge for our community lies in creating quality data sets necessary to derive benefit from these emerging analytical approaches.

### Conclusions

Using machine learning and patient-reported experience, 3 groups of patients with DCM were defined. These groups differed in the number of clinical features reported but not in the severity of DCM, time since diagnosis, or time with DCM. The significance and generalization of this study remain uncertain. Overall, this study confirms the role of machine learning in DCM research, but more pressingly, it confirms the need to curate the right data sets.

## Appendix

Multimedia Appendix 1 Additional material regarding survey completion.

## Abbreviations

DCM: degenerative cervical myelopathy

## Data Availability

The data sets generated and analyzed during this study are available from the corresponding author upon reasonable request.
