# Supplementary material for: Machine Learning and Symptom Patterns in Degenerative Cervical Myelopathy: Web-Based Survey Study
Source: JMIR Form Res. 2024 Jan 25;8:e54747. doi: 10.2196/54747 (PMC10853854; doi:10.2196/54747)
Supplement: Multimedia Appendix 1 [file formative_v8i1e54747_app1.docx]

**Supplementary Materials**

**Supplementary Table 1.** Table showing the extent of Survey Completion

| **Survey Completion Description** | **Frequency** |
| --- | --- |
| Full survey completion: Demographics, mJOA, current symptoms, first symptom, fibromyalgia, current effects on life, first effect on life and category satisfaction questions answered | 165 |
| Demographics, mJOA, current symptoms, first symptom, fibromyalgia, current effects on life and first effect on life questions answered | 2 |
| Demographics, mJOA, current symptoms, first symptom, fibromyalgia and current effects on life questions answered (effects on life questions only considered answered if at least 1 effect is reported) | 2 |
| Demographics, mJOA, current symptoms, first symptom and fibromyalgia questions answered | 2 |
| Demographics, mJOA and current symptoms questions answered (symptoms questions only considered answered if at least 1 symptom is reported) | 2 |
| Demographics and mJOA questions answered | 8 |
| Only demographics questions answered | 2 |
| No questions answered or answered “No” to either “Do you suffer from myelopathy (disease of the spinal cord)?” or "Is your myelopathy caused by any of the conditions encompassed by the term Degenerative Cervical Myelopathy*?" | 6 |
| Total | 189 |

**Supplementary Table 2.** Table expanding on the acronyms used

| **Survey.question** | **Survey.code** |
| --- | --- |
| Hand.shaking | HS |
| Reduced.grip.strength | RGS |
| Reduced.dexterity..less.able.to.perform.complex.tasks.with.your.hands. | RD |
| Muscle.spasms.or.twitches..in.your.arms. | MSA |
| Leg.shaking | LS1 |
| Heavy.legs | HL |
| Dragging.legs | DL |
| Muscle.spasms.or.twitches..in.your.legs. | MSL |
| Falls | F1 |
| Lack.of.control.of.legs | LCL |
| Clumsiness | C |
| Difficulty.emptying.bladder | DEB |
| Urinary.incontinence | UI |
| Faecal.incontinence | FI |
| Symptom.variability.day.by.day | SVD |
| Symptom.variability.hour.by.hour | SVH |
| Insomnia | I |
| Waking.to.go.to.the.toilet | W |
| Difficulty.breathing.when.performing.physical.activity | DBPA |
| Difficulty.breathing.when.lying.flat | DBLF |
| Hot.flushes.and.or.sweating | HF |
| Arm.numbness | AN |
| Hand.numbness | HN |
| Leg.numbness | LN |
| Pins.and.needles.in.your.hand | PNH |
| Pins.and.needles.in.your.arm | PNA |
| Pins.and.needles.in.your.leg | PNL |
| Neck.pain | NP |
| Arm.pain | AP |
| Leg.pain | LP |
| Back.pain | BP |
| Neck.stiffness | NS |
| Arm.stiffness | AS |
| Leg.stiffness | LS2 |
| Neck.clicking | NC |
| Depression.low.mood | D |
| Anxiety | A |
| Impaired.cognition | IC |
| Fatigue | F |
| Headache | H |
| Poor.balance | PB |
| Shoulder.pain | SP |
| Electric.shock.like.sensations.down.your.back..Lhermitte.s.sign. | LS3 |
| Dizziness | DZ |
| Altered.temperature.sensation | AT |
| Pain.from.previously.non.painful.sensations..Allodynia. | ALL |
| Choking.swallowing.problems | CK |
| Face.pain | FP |
| Face.numbness | FN |
| Constipation | CO |
| Eyesight.problems | EP |
| Ringing.in.your.ears..Tinnitus. | TNN |
| Abdominal.pain | ABP |
| Nausea…vomiting | NU |
| Total.number.of.symptoms.reported | TNSR |
| Falls.1 | F2 |
| Unable.to.drive | U2D |
| Unable.to.exercise | U2E |
| Difficulties.climbing.stairs | DCS |
| Unable.to.get.up.out.of.a.chair | U2GC |
| Unable.to.get.out.of.bed | U2GB |
| Muscle.weakness | MW |
| Reduced.walking.distance | RWD |
| Reduced.activity.such.that.you.are.unable.to.have.fun | R2F |
| Reduced.activity.such.that.you.are.unable.to.work | R2W |
| Difficulty.with.travel | DWT |
| Difficulty.with.planning.life | DWL |
| Inability.to.turn.over.in.bed | I2TB |
| Unable.to.get.comfortable.in.bed | U2CB |
| Difficulties.with.social.interaction | DWSI |
| Financial.difficulties | FD |
| Difficulty.parenting.and.in.family.life | D2P |
| Lifting.heavy.objects.is.difficult.painful | LIP |
| Reduced.sex.life | RSL |
| Difficulty.thinking.concentrating.memory.problems | D2C |
| Difficulty.in.performing.household.tasks | D2T |
| Living.in.fear.of.damaging.spinal.cord.further.from.falls.trauma | TRU |
| Total.number.of.effects.on.life.reported | TNER |
